# The Association between Ambient Temperature and Childhood Hand, Foot, and Mouth Disease in Chengdu, China: A Distributed Lag Non-linear Analysis

**DOI:** 10.1038/srep27305

**Published:** 2016-06-01

**Authors:** Fei Yin, Tao Zhang, Lei Liu, Qiang Lv, Xiaosong Li

**Affiliations:** 1 West China School of Public Health, Sichuan University, Chengdu, Sichuan, People’s Republic of China; 2 Sichuan Center for Disease Control and Prevention, Chengdu, Sichuan, People’s Republic of China

## Abstract

Hand, foot and mouth disease (HFMD) has recently been recognized as a critical challenge to disease control and public health response in China. This study aimed to quantify the association between temperature and HFMD in Chengdu. Daily HFMD cases and meteorological variables in Chengdu between January 2010 and December 2013 were obtained to construct the time series. A distributed lag non-linear model was performed to investigate the temporal lagged association of daily temperature with age- and gender-specific HFMD. A total of 76,403 HFMD cases aged 0–14 years were reported in Chengdu during the study period, and a bimodal seasonal pattern was observed. The temperature-HFMD relationships were non-linear in all age and gender groups, with the first peak at 14.0–14.1 °C and the second peak at 23.1–23.2 °C. The high temperatures had acute and short-term effects and declined quickly over time, while the effects in low temperature ranges were persistent over longer lag periods. Males and children aged <1 year were more vulnerable to temperature variations. Temperature played an important role in HFMD incidence with non-linear and delayed effects. The success of HFMD intervention strategies could benefit from giving more consideration to local climatic conditions.

Hand, foot and mouth disease (HFMD) is an emerging viral infectious disease mainly occurring in children under the age of 5. It is mainly caused by enterovirus 71 (EV71) and coxsackievirus A16 (Cox A16)^1^,^2^. Most patients show self-limiting illness typically including fever and vesicular exanthema on hands, feet, mouth, and buttocks^3^. However, sometimes serious neurological and cardiopulmonary complications may occur^4^.

In recent years, HFMD epidemics are frequent and widespread in the Asia-Pacific region, especially in China, Japan, Malaysia and Singapore^5^,^6^,^7^,^8^. According to the national surveillance system of China, over 4.5 million cases has been reported from January 2013 to December 2014. The epidemic of HFMD has become a serious public health problem in China. At present, there is no effective chemoprophylaxis or vaccination available for HFMD. Therefore, epidemiological surveillance of HFMD is essential for health officials to take proper and timely interventions to prevent and control the disease.

The incidence of HFMD has been reported to exhibit seasonal variation in a number of different regions^6^,^7^,^8^,^9^,^10^. For instance, epidemic peaks were observed in summer and late autumn^11^,^12^. The observed seasonality suggested that the incidence of HFMD might be influenced by temperature. Several studies have been conducted to explore the relationship between temperature and HFMD with inconsistent findings reported, and the nature and extent of the relationship remains highly controversial. For example, many studies found that temperature was positively related to the incidence of HFMD^12^,^13^,^14^, while a study in Taiwan reported a negative association between temperature and HFMD when the temperature is above 26 °C.

Two aspects of the temperature effects deserve special attention: the non-linear and lag characteristics. On one side, previous studies have provided evidence of the nonlinear relationship between temperature and HFMD^13^,^14^. On the other side, some time series studies have reported the delayed effect of temperature exposure on HFMD^11^,^12^. However, they typically used a single fixed lag model to measure the moving average lag effects which may result in an underestimation of the effect of temperature on HFMD.

The association between temperature and HFMD is complex due to the two characteristics mentioned above. Existing inconsistent findings may be due to two reasons. Firstly, different areas may have different relationship patterns because of regional variations. Secondly, the misspecification of the single fixed lag and/or the invalid assumption of the linear relationship might lead to biased estimates. However, there are few studies regarding the pattern of delayed effect of temperature after accounting for the nonlinearity.

The current study aimed to explore the lag association between temperature and HFMD. Specifically, a distributed lag non-linear model (DLNM) was used to examine the temporal lagged association between daily temperature and daily HFMD cases using the data from 2010 to 2013 in Chengdu, China. DLNM has been developed to estimate simultaneously non-linear and delayed effects. Using this more reasonable model, a better understanding might be obtained for the association between temperature and HFMD. Also, the result may provide information on appropriate allocation of public health resources for better disease control and prevention.

## Results

**Figure 1:**
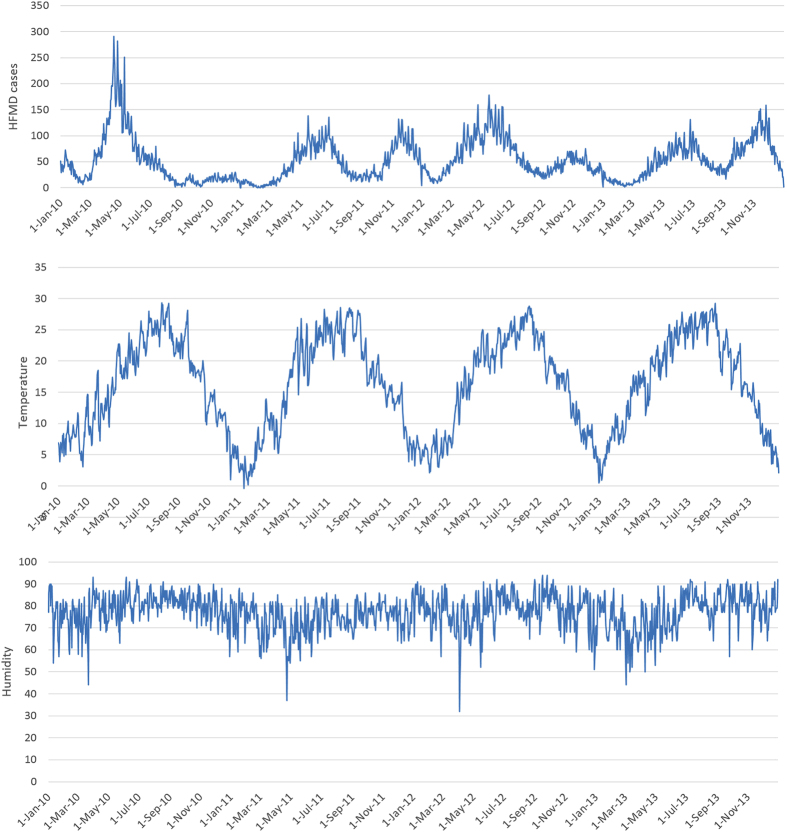
Daily distribution of meteorological variables and HFMD cases in Chengdu, 2010–2013.

Between 1 January 2010 and 31 December 2013, a total of 76,403 HFMD cases aged 0–14 years were reported in Chengdu. Children aged 0–5 years were majority of the victims, which accounted for 97.18% (74,247 cases) of all reported cases over the study period. Of 76,403 HFMD cases, 45,771 were males and 30,632 were females, with a male-to-female sex ratio 1.49. Descriptive statistics for daily HFMD cases and meteorological variables was shown in Table 1. There was an average of 52.3 daily HFMD cases over the study period. The mean level of daily mean temperature and relative humidity were 16.21 °C and 76.96%, respectively. Figure 1 depicted the time series of daily HFMD cases and meteorological variables during the study period, indicating a seasonal pattern. The seasonal peak of HFMD cases was found to differ from year to year, although it typically occurred during the summer and winter months. The first seasonal peak occurred between April to July, followed by the second peak from October to December.

**Figure 2:**
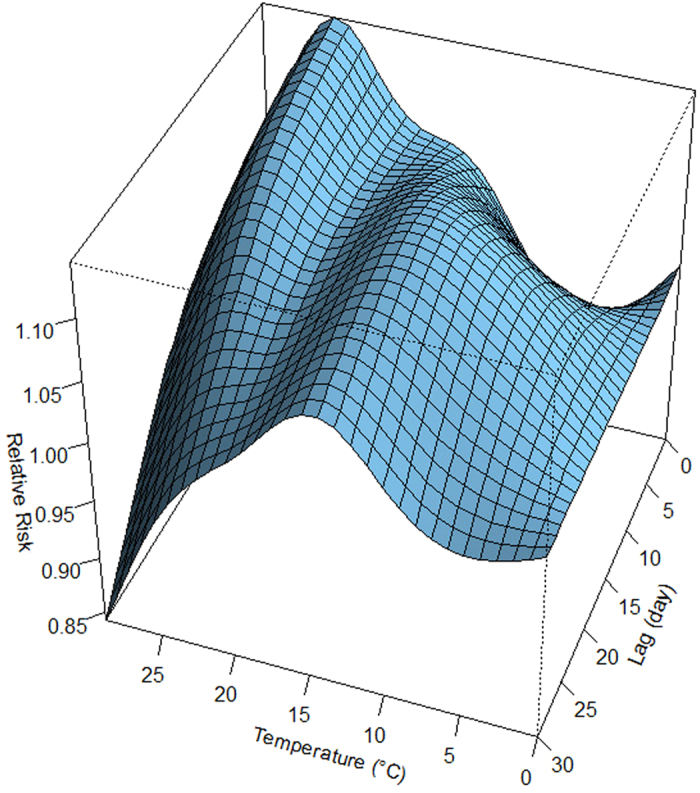
Three-dimensional plot of the relationship between mean temperature and HFMD over 30 lag days.

**Figure 3:**
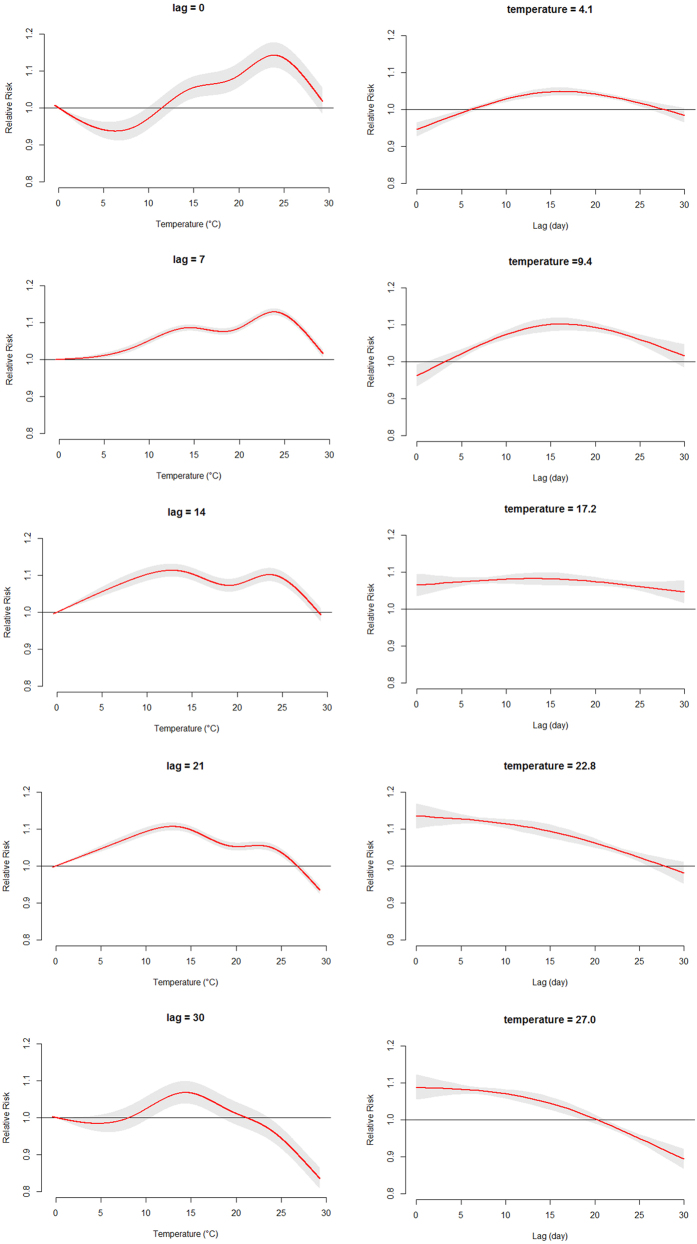
Plot of relative risk (RR) by temperature at specific lags (left), and RR by lag at specific temperatures (right).

Figure 2 showed the three-dimensional relationship between daily mean temperature and HFMD cases along 30 lag days. Overall, the estimated effects of temperature on HFMD incidence were non-linear, with larger relative risk (RR) at the high temperature. Figure 3 illustrated the relative risk by temperature at specific lags (0, 7, 14, 21 and 30) and by lag at specific temperatures (4.1, 9.4, 17.2, 22.8 and 27.0 °C), corresponding approximately to 5th, 25th, 50th, 75th and 95th percentiles of temperature distribution. It was found that the high temperatures had acute and short-term effects and declined quickly over time, while the effects in low temperature ranges were persistent over longer lag periods. For instance, the high temperature (27.0 °C) had the maximum RR for HFMD cases on the current day, which decreased quickly during the following days. However, the low temperature (4.1 °C) had the minimum RR on the current day and had the maximum RR at lag 16 days, which decreased slowly during the following days. It suggested that the relationship between temperature and HFMD might have a different lag pattern.

**Figure 4:**
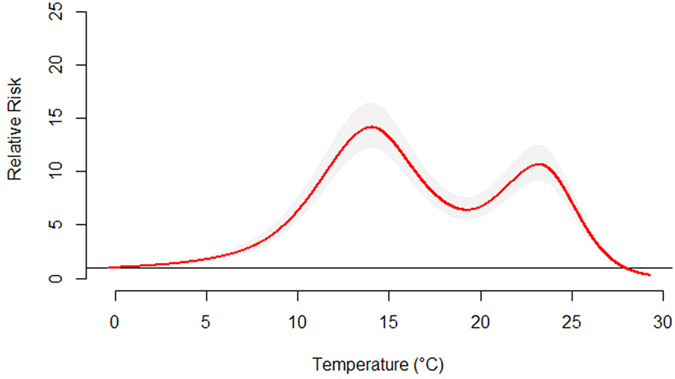
The overall relative risks of mean temperature for total HFMD cases over 30 days.

**Figure 5:**
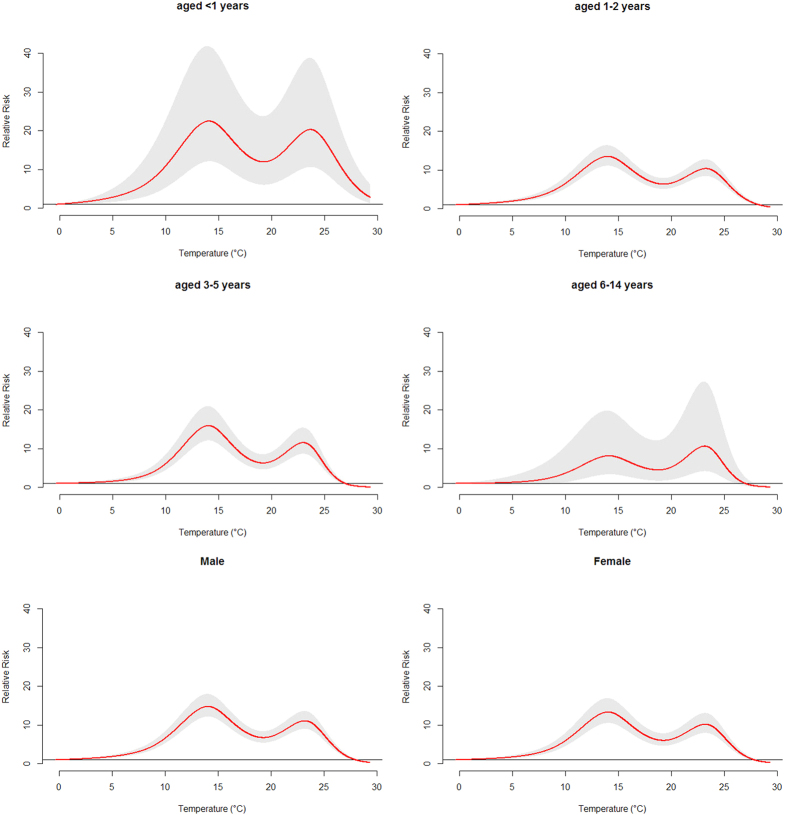
The overall relative risks of mean temperature for age- and gender-specific HFMD cases over 30 days.

Figure 4 demonstrated the overall effect of temperature, summing up the contributions for the 30 days of lag. The exposure-response curve was an approximately M-shape. The RR increased with the increment of temperature and it reached the first peak at 14.0 °C and then turned to decrease. Again, the RR began to increase when then temperature was above 19.3 °C. It reached the second smaller peak at 23.2 °C and then began to decrease. Figure 5 presented the RR of cumulative exposure to mean temperature over 30 days for age- and gender-specific HFMD cases. For males and females, the RR followed the similar trends as the total RR. For these two groups, the RR reached the first peak at 14.0 °C and reached the second smaller peak at 23.2 °C. For children aged <1, 1–2 and 3–5 years, the RR also followed the similar trends as the total RR. For children aged <1 year, the RR reached the first peak at 14.1 °C and reached the second smaller peak at 23.7 °C. For children aged 1–2 years, the RR reached the first peak at 14.0 °C and reached the second smaller peak at 23.3 °C. For children aged 3–5 years, the RR reached the first peak at 14.0 °C and reached the second smaller peak at 23.0 °C. For children aged 6–14 years, there was a little difference. The RR reached the first peak at 14.1 °C and reached the second larger peak at 23.1 °C.

The risks of different mean temperatures for total, gender- and age-specific HFMD cases along the lags were summarized in Table 2. The effects of mean temperature on childhood HFMD differed between males and females. We found that mean temperature had the higher risk estimates of HFMD incidence in males than in females. For male, the highest RR value was 14.76 at lag0–30. While for female, the highest RR value was 13.31 at lag0–30. For age-specific RRs, mean temperature had the highest risk estimates of HFMD incidence in children aged <1 year. For children aged <1 year, the highest RR value was 22.47 at lag0–30. For children aged 1–2 and 3–5 years, the highest RR value were 13.46 and 15.89 separately at lag0–30. For children aged 6–14 years, the highest RR value was 10.60 at lag0–30.

In the sensitivity analyses, when the df for climate variables were varied between 4 and 7, similar results were obtained.

## Discussion

In this study, we examined the association of temperature with age- and gender-specific HFMD among children in Chengdu, China, during 2010–2013. A distributed lag non-linear model was performed to investigate the temporal lagged association of daily temperature with HFMD. This is the first research targeted at the non-linear associations of delayed and cumulative exposure to temperature with age- and gender-specific HFMD among children in southwest China.

In our study, the descriptive analysis of temporal trends showed that there were two peaks per year. The first peak occurred in the late spring, along with the second peak in early winter. These seasonal patterns were similar to those found in other areas of China (Shangxi, Guangxi and Guangdong Province) as well as many other countries (Malaysia, Singapore, UK)^6^,^7^,^9^,^11^,^12^,^15^. However, there are different seasonal patterns in other regions. For instance, in Finland, most HFMD cases were reported in autumn^10^. In Japan, one single peak was observed in July 2011^8^. This inconsistent might due to various environmental factors such as climatic, geographic, social-economic factors, etc.

The age-specific results indicated that children aged <1 year appeared to be most vulnerable to temperature changes. A previous study has indicated that the level of maternal antibodies to EV71 in the infants waned one month after birth^16^. The lack of immunity might increase the susceptivity of children less than 1 year of age to the temperature variations. The gender-specific results showed that that males were more vulnerable to temperature effect than females, which indicated that susceptibility might be distinct at the host genetic level^17^. In addition, boys are generally more active than girls, which make boys have more chances of being exposed to enteroviruses and are more easily infected by HFMD.

In this study, DLNM was used to examine the relationship between temperature and HFMD. The result indicated that temperature had a non-linear relationship with the HFMD incidence. An interesting finding was that the exposure-response curve was an approximately M-shape. This was consistent with the descriptive analysis of temporal trends in which the number of reported HFMD cases displayed a bimodal pattern (peaked twice a year). Although the exact mechanism remains unclear, this may be partly due to the fact that temperature could influence the survival and transmission of the HFMD virus, as well as children’s activity and behavior, and thus affect the dynamics of disease transmission. An experimental study found that the stability of enteric viruses was generally influenced by environmental factors such as temperature and relative humidity^18^. In addition, children’s activity could be influenced by temperature. Children are more likely to play outside or swim during summer. Previous studies have indicated that the summer peaks of enteric viruses were associated with swimming^19^,^20^,^21^. However, higher temperature might reduce the outdoor activities of children, and thus decrease their contacts with other children^16^. While in winter, children usually stay indoor longer which may increase their contacts with household members and result in higher transmission. Further study is needed to investigate the underlying mechanism of this complex association.

Most previous studies examined the effect of temperature on HFMD at a weekly or monthly scale. Our study suggested that it might be more reasonable to do the research based on daily data, which could provide more detailed information. Furthermore, weekly or monthly data rather than daily data may underestimate the association between weather factors and HFMD incidence, which may affect the accuracy of exposure assessment. In addition, the analysis at a daily scale might be more suitable for the prevention and control of HFMD by providing more timely information. Therefore, the local health authorities could have enough time to prepare for the possible upcoming HFMD epidemic.

The limitations of this study should be acknowledged: 1) Our study was based on data collected from only one city. Therefore, the results from this study might not be generalized to other regions with different temperature zone. Multi-city studies in different geographical locations are needed for further research. 2) We could not differentiate the pathogens of HFMD cases reported to the CISDCP system. Therefore, we were unable to investigate the specific impacts of temperature on different pathogens. 3)

In conclusion, our study revealed a non-linear relationship between daily temperature and HFMD. The exposure-response curve was an approximately M-shape. Children aged <1 year and male children were more vulnerable to the temperature variations. Our study provides information to better understand the effect of temperature variation on HFMD and may have policy implications for disease prevention and control. The success of HFMD intervention strategies could benefit from giving more consideration to local climate conditions.

## Materials and Methods

### Study area

Chengdu is the provincial capital of Sichuan province in southwest China, as well as a major city in western China. According to the 2010 census, Chengdu is the fifth-most populous city in China. Chengdu covers an area of 12,132 km^2^, with a population of approximately 14 million people. It has a monsoon-influenced humid subtropical climate and is largely mild and humid.

### Data sources

Daily data of reported HFMD cases in Sichuan province from 2010 to 2013 were obtained from the China Information System for Disease Control and Prevention (CISDCP). HFMD was classified as a class “C” notifiable disease by the Ministry of Health of China, which demands that all HFMD cases should be reported to CISDCP within 24 hours after diagnosis^22^. In this study, all HFMD cases were confirmed according to the unified diagnostic criteria issued by the Ministry of Health of China 22. A recent data quality survey report has showed that the reported HFMD data are of good quality, with reporting completeness of 99.84% and accuracy of the information reported to be 92.76%^23^. The HFMD cases aged 0–14 years accounting for 99.68% of all the HFMD cases were chosen in this study.

Meteorological data including daily mean temperature, relative humidity and rainfall of Chengdu were collected from China Meteorological Data Sharing Service System (http://cdc.cma.gov.cn).

### Data analysis

DLNM represent a modelling framework to describe simultaneously non-linear and delayed effects^24^. A Poisson generalized linear regression combined with DLNM was applied to explore the effect of temperature on HFMD incidence. The model was specified as:


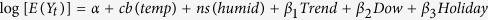


where 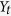 is the reported daily HFMD case counts on day t; 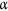
is the intercept; cb(temp) indicates the “cross-basis” function, which is obtained by DLNM to model non-linear and distributed lag effects of temperature. ns means a smooth function based on natural cubic spline. A natural cubic spline DLNM was used to model the nonlinear association between temperature and HFMD. Akaike Information Criterion (AIC) was adopted to choose the df for temperature and lag^24^. The final composition of the function was a natural cubic spline of temperature with five df and a natural cubic spline with three df for lag days. Previous studies specified the lagged effect of temperature from 13 days to 6 weeks of lag^14^,^25^,^26^,^27^. In this study, we found the effect of temperature on childhood HFMD was negligible for lags above 30 days, so a maximum lag of 30 days was used to explore the potential lag associations. Three df was used to smooth humidity (humid) and rainfall (rain)^26^,^27^. Trend is a variable of the year and calendar month used to control for seasonality and long term trend. Dow is day of the week on day t. Holiday is a binary variable which is “1” if day t was a public holiday. 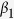, 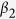 and 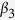 are the regression coefficients. Rainfall was insignificantly associated with HFMD, therefore the variable was removed from the final model.

In our study, the lowest temperature (0 °C) was used as the reference value to calculate the relative risk. It is important to note that the change of the reference value may affect the value of the relative risk and the width of confidence interval, but it will not affect the RR curve itself.

Stratified analyses were performed by gender (male and female) and age group (<1, 1–2, 3–5 and 6–14 years). The age groups were devided based on the different daily activity and environment. In China, children aged 0–2 years are usually cared at home. But children aged <1 year are different from those aged 1–2 years in terms of daily activities. The group aged 3–5 years usually attend kindergartens. And children over 6 years of age go to school. Sensitive analyses were conducted to test the robustness of our results: varying the df (4–7) for climate variables. The “dlnm” and “spline” packages in the R software (version 3.1.1) was used to create the DLNM model.

## Additional Information

**How to cite this article**: Yin, F. *et al*. The Association between Ambient Temperature and Childhood Hand, Foot, and Mouth Disease in Chengdu, China: A Distributed Lag Non-linear Analysis. *Sci. Rep.* **6**, 27305; doi: 10.1038/srep27305 (2016).

## Figures and Tables

**Table 1:** Descriptive statistics for daily HFMD cases and meteorological variables in Chengdu, 2010–2013.

	Mean	SD	Min.	Median	Max.
Cases					
HFMD cases	52.30	40.31	0	44.00	291
HFMD cases in male	31.54	24.25	1	26.00	170
HFMD cases in female	21.29	16.63	1	18.00	122
HFMD cases aged <1 year	4.69	3.57	1	4.00	27
HFMD cases aged 1–2 years	30.01	21.92	1	26.00	158
HFMD cases aged 3–5 years	17.49	16.19	1	13.00	117
HFMD cases aged 6–14 years	2.35	1.70	1	2.00	12
Meteorological variable					
Mean temperature ( °C)	16.21	7.49	−0.40	17.20	29.30
Relative humidity (%)	76.96	8.11	32.00	78.00	94.00

**Table 2:** The relative risks of different temperatures for age-specific and gender-specific HFMD cases in Chengdu.

	Temperature( °C)^a^	RR(95%CI)		
		Lag0	Lag14	Lag0–30(overall effect)
total	4.1	0.95(0.93,0.97)	1.05(1.04,1.06)	1.55(1.37,1.76)
	14.0^b^	1.04(1.02,1.07)	1.11(1.09,1.13)	14.14(12.15,16.46)
	17.2	1.06(1.03,1.10)	1.08(1.07,1.10)	8.42(7.17,9.88)
	23.2^b^	1.14(1.11,1.17)	1.10(1.08,1.12)	10.66(9.08,12.51)
	27.0	1.09(1.06,1.13)	1.05(1.04,1.07)	2.05(1.73,2.43)
male	4.1	0.96(0.94,0.98)	1.04(1.03,1.05)	1.59(1.35,1.87)
	14.0^b^	1.07(1.03,1.11)	1.10(1.08,1.12)	14.76(12.12,17.97)
	17.2	1.09(1.04,1.13)	1.08(1.05,1.10)	8.82(7.17,10.86)
	23.2^b^	1.15(1.11,1.20)	1.10(1.07,1.12)	11.03(8.96,13.57)
	27.0	1.10(1.06,1.15)	1.05(1.03,1.07)	2.14(1.72,2.67)
female	4.1	0.93(0.90,0.96)	1.05(1.04,1.07)	1.50(1.23,1.82)
	14.0^b^	1.01(0.97,1.05)	1.12(1.10,1.15)	13.31(10.48,16.90)
	17.2	1.04(0.99,1.08)	1.09(1.06,1.12)	7.87(6.11,10.12)
	23.2^b^	1.12(1.07,1.17)	1.11(1.08,1.13)	10.16(7.90,13.06)
	27.0	1.08(1.02,1.13)	1.06(1.03,1.09)	1.92(1.47,2.51)
<1 year	4.1	1.02(0.94,1.10)	1.03(0.99,1.07)	2.29(1.39,3.78)
	14.1^b^	1.15(1.03,1.29)	1.09(1.03,1.16)	22.47(12.08,41.78)
	17.2	1.19(1.06,1.34)	1.07(1.00,1.14)	14.85(7.76,28.44)
	23.7^b^	1.29(1.13,1.44)	1.06(1.00,1.13)	20.28 (10.60,38.82)
	27.0	1.31(1.16,1.48)	1.03(0.97,1.10)	8.82(4.50,17.29)
1–2 years	4.1	0.99(0.97,1.02)	1.03(1.02,1.04)	1.67(1.42,1.96)
	14.0^b^	1.11(1.06,1.15)	1.09(1.07,1.11)	13.46(11.06,16.38)
	17.2	1.12(1.08,1.17)	1.06(1.04,1.09)	8.18(6.64,10.06)
	23.3^b^	1.18(1.14,1.23)	1.08(1.06,1.11)	10.33(8.40,12.71)
	27.0	1.15(1.10,1.20)	1.04(1.02,1.06)	2.47(1.98,3.07)
3–5 years	4.1	0.86(0.83,0.89)	1.08(1.06,1.10)	1.35(1.08,1.69)
	14.0^b^	0.93(0.88,0.97)	1.16(1.12,1.19)	15.89(12.07,20.93)
	17.2	0.94(0.89,0.99)	1.12(1.09,1.16)	8.54(6.39,11.41)
	23.0^b^	1.03(0.98,1.09)	1.15(1.12,1.19)	11.51(8.62,15.38)
	27.0	0.93(0.88,0.98)	1.08(1.05,1.12)	0.91(0.67,1.25)
6–14 years	4.1	0.88(0.78,0.99)	1.05(0.99,1.12)	1.15(0.55,2.42)
	14.1^b^	0.98(0.83,1.16)	1.08(0.99,1.19)	8.10(3.32,19.76)
	17.2	1.06(0.89,1.26)	1.05(0.96,1.16)	5.21(2.03,13.38)
	23.1^b^	1.23(1.03,1.46)	1.07(0.97,1.18)	10.60(4.12,27.30)
	27.0	1.15(0.96,1.38)	1.03(0.93,1.14)	0.94(0.34,2.58)

^a^4.1 °C, 17.2 °C and 27.0 °C represented the 5^th^ percentile, 50^th^ percentile and 95th percentile of temperature in Chengdu, respectively.

^b^The two peak values of temperature for total children, gender-specific and age-specific HFMD cases, respectively.

## References

[b1] Xing W. . Hand, foot, and mouth disease in China, 2008–12: an epidemiological study. Lancet Infect Dis 14, 308–318 (2014). 24485991 10.1016/S1473-3099(13)70342-6 PMC4035015

[b2] Gopalkrishna V., Patil P. R., Patil G. P. & Chitambar S. D. Circulation of multiple enterovirus serotypes causing hand, foot and mouth disease in India. J Med Microbiol 61, 420–425 (2012). 22052995 10.1099/jmm.0.036400-0

[b3] Jiang M. . Autopsy findings in children with hand, foot, and mouth disease. N Engl J Med 367, 91–92 (2012). 22762340 10.1056/NEJMc1110981

[b4] Ooi M. H., Wong S. C., Lewthwaite P., Cardosa M. J. & Solomon T. Clinical features, diagnosis, and management of enterovirus 71. Lancet Neurol 9, 1097–1105 (2010). 20965438 10.1016/S1474-4422(10)70209-X

[b5] Ni H. . Epidemiological and etiological characteristics of hand, foot, and mouth disease in Ningbo, China, 2008–2011. J Clin Virol 54, 342–348 (2012). 22652041 10.1016/j.jcv.2012.04.021

[b6] Ang L. W. . Epidemiology and control of hand, foot and mouth disease in Singapore. Ann Acad Med Singap 38, 106–112 (2009). 19271036

[b7] Chua K. B. & Kasri A. R. Hand foot and mouth disease due to enterovirus 71 in Malaysia. Virol Sin 26, 221–228 (2011). 21847753 10.1007/s12250-011-3195-8 PMC8222466

[b8] Fujimoto T. . Hand, foot, and mouth disease caused by coxsackievirus A6, Japan, 2011. Emerg Infect Dis 18, 337–339 (2012). 22304983 10.3201/eid1802.111147 PMC3310456

[b9] Bendig J. & Fleming D. Epidemiological, virological, and clinical features of an epidemic of hand, foot, and mouth disease in England and Wales. Commun Dis Rep CDR Rev 6, R81–86 (1996). 8664928

[b10] Blomqvist S. . Co-circulation of coxsackieviruses A6 and A10 in hand, foot and mouth disease outbreak in Finland. J Clin Virol 48, 49–54 (2010). 20189452 10.1016/j.jcv.2010.02.002

[b11] Song Y. . Time Series Analyses of Hand, Foot and Mouth Disease Integrating Weather Variables. PLoS One 10, e0117296 (2015). 25729897 10.1371/journal.pone.0117296 PMC4346267

[b12] Wei J. . The Effect of Meteorological Variables on the Transmission of Hand, Foot and Mouth Disease in Four Major Cities of Shanxi Province, China: A Time Series Data Analysis (2009–2013). PLoS Negl Trop Dis 9, e0003572 (2015). 25742504 10.1371/journal.pntd.0003572 PMC4351101

[b13] Huang Y. . Effect of meteorological variables on the incidence of hand, foot, and mouth disease in children: a time-series analysis in Guangzhou, China. BMC Infect Dis 13, 134 (2013). 23497074 10.1186/1471-2334-13-134 PMC3626782

[b14] Onozuka D. & Hashizume M. The influence of temperature and humidity on the incidence of hand, foot, and mouth disease in Japan. Sci Total Environ 410, 119–125 (2011). 22014509 10.1016/j.scitotenv.2011.09.055

[b15] Xie Y., Chongsuvivatwong V., Tang Z., McNeil E. B. & Tan Y. Spatio-temporal clustering of hand, foot, and mouth disease at the county level in Guangxi, China. PLoS One 9, e88065 (2014). 24505378 10.1371/journal.pone.0088065 PMC3914922

[b16] Ooi E.-E., Phoon M.-C., Ishak B. & Chan S.-H. Seroepidemiology of human enterovirus 71, Singapore. Emerg Infect Dis 8, 995–997 (2002). 12194783 10.3201/eid0809.10.3201/eid0809.010397 PMC2732542

[b17] Chen T.-C. . Combining multiplex reverse transcription-PCR and a diagnostic microarray to detect and differentiate enterovirus 71 and coxsackievirus A16. J Clin Microbiol 44, 2212–2219 (2006). 16757623 10.1128/JCM.02393-05 PMC1489440

[b18] Abad F. X., Pinto R. M. & Bosch A. Survival of enteric viruses on environmental fomites. Appl Environ Microbiol 60, 3704–3710 (1994). 7986043 10.1128/aem.60.10.3704-3710.1994 PMC201876

[b19] Bashiardes S. . Analysis of enterovirus and adenovirus presence in swimming pools in Cyprus from 2007–2008. Water Sci Technol 63, 2674–2684 (2011). 22049764 10.2166/wst.2011.484

[b20] D’alessio D. J., Minor T. E., Allen C. I., Tsiatis A. A. & Nelson D. B. A study of the proportions of swimmers among well controls and children with enterovirus-like illness shedding or not shedding an enterovirus. Am J Epidemiol 113, 533–541 (1981). 7223734 10.1093/oxfordjournals.aje.a113129

[b21] Hawley H., Morin D., Geraghty M., Tomkow J. & Phillips C. Coxsackievirus B epidemic at a Boy’s Summer Camp. Isolation of virus from swimming water. JAMA 226, 33–36 (1973). 4801640

[b22] The Ministry of Health of China. Hand, Foot and Mouth Disease Prevention and Control Guideline, China. (2009) Available at :http://www.gov.cn/gzdt/2009-06/04/content_1332078.htm. (Accessed: 4th October 2015).

[b23] Ji Y., Guo Y., Guo Q., Zhang C. & JQ M. Analysis of notifiable infectious disease surveillance and reporting of medical units in 6 provinces. Mod Prev Med 38, 4266–4268 (2011).

[b24] Gasparrini A., Armstrong B. & Kenward M. Distributed lag non-linear models. Stat Med 29, 2224–2234 (2010). 20812303 10.1002/sim.3940 PMC2998707

[b25] Chen C. . Short-term effects of meteorological factors on children hand, foot and mouth disease in Guangzhou, China. Int J Biometeorol 58, 1605–1614 (2014). 24258319 10.1007/s00484-013-0764-6

[b26] Xu M. . Non-Linear Association between Exposure to Ambient Temperature and Children’s Hand-Foot-and-Mouth Disease in Beijing, China. PLoS One 10, e0126171 (2014). 26010147 10.1371/journal.pone.0126171 PMC4444089

[b27] Zhu L. . The Impact of Ambient Temperature on Childhood HFMD Incidence in Inland and Coastal Area: A Two-City Study in Shandong Province, China. Int J Environ Res Public Health 12, 8691–8704 (2015). 26213955 10.3390/ijerph120808691 PMC4555242

